# Enhanced Spontaneous Antibacterial Activity of δ-MnO_2_ by Alkali Metals Doping

**DOI:** 10.3389/fbioe.2021.788574

**Published:** 2022-01-04

**Authors:** Yali Yan, Ning Jiang, Xin Liu, Jie Pan, Mai Li, Chunrui Wang, Pedro H. C. Camargo, Jiale Wang

**Affiliations:** ^1^ College of Science, Donghua University, Shanghai, China; ^2^ Department of Oral and Craniomaxillofacial Science, Shanghai Key Laboratory of Stomatology, College of Stomatology, Ninth People’s Hospital, Shanghai Jiao Tong University School of Medicine, Shanghai, China; ^3^ Department of Dental Materials, Shanghai Key Laboratory of Stomatology, Shanghai Biomaterials Research and Testing Center, National Center for Stomatology, National Clinical Research Center for Oral Diseases, Shanghai Ninth People’s Hospital, Shanghai Jiao Tong University School of Medicine, College of Stomatology, Shanghai Jiao Tong University, Shanghai, China; ^4^ Department of Orthodontics, Shanghai Stomatological Hospital, Fudan University, Shanghai, China; ^5^ Shanghai Key Laboratory of Craniomaxillofacial Development and Diseases, Fudan University, Shanghai, China; ^6^ Department of Chemistry, University of Helsinki, Helsinki, Finland; ^7^ Shanghai Institute of Intelligent Electronics and Systems, Donghua University, Shanghai, China

**Keywords:** MnO_2_, doping, antibacterial property, reactive oxygen species, alkali metal ions

## Abstract

Recently, the widespread use of antibiotics is becoming a serious worldwide public health challenge, which causes antimicrobial resistance and the occurrence of superbugs. In this context, MnO_2_ has been proposed as an alternative approach to achieve target antibacterial properties on *Streptococcus* mutans (S. mutans). This requires a further understanding on how to control and optimize antibacterial properties in these systems. We address this challenge by synthesizing δ-MnO_2_ nanoflowers doped by magnesium (Mg), sodium (Na), and potassium (K) ions, thus displaying different bandgaps, to evaluate the effect of doping on the bacterial viability of S. mutans. All these samples demonstrated antibacterial activity from the spontaneous generation of reactive oxygen species (ROS) without external illumination, where doped MnO_2_ can provide free electrons to induce the production of ROS, resulting in the antibacterial activity. Furthermore, it was observed that δ-MnO_2_ with narrower bandgap displayed a superior ability to inhibit bacteria. The enhancement is mainly attributed to the higher doping levels, which provided more free electrons to generate ROS for antibacterial effects. Moreover, we found that δ-MnO_2_ was attractive for *in vivo* applications, because it could nearly be degraded into Mn ions completely following the gradual addition of vitamin C. We believe that our results may provide meaningful insights for the design of inorganic antibacterial nanomaterials.

## Introduction

Manganese oxides (MnO_2_) have been extensively studied due to the structural multiformity. The various structures, corresponding to different chemical and physical properties, have been widely applied in catalysis, batteries, sensors, molecular sieves, energy storage, etc. (Dawadi et al., 2020; Ye et al., 2020; Marciniuk et al., 2021; Ouyang et al., 2021; Wang et al., 2021). Particularly, δ-MnO_2_ has attracted considerable attention due to its unique layered structure, where its bandgap can be tuned by filling ions between the layers (Luo et al., 2000; Wang et al., 2017). Herein, δ-MnO_2_ samples with different bandgaps, doped by alkali metals such as magnesium (Mg), sodium (Na), or potassium (K), has been used as antibacterial materials.

Recently, the abuse of antibiotics is becoming a serious worldwide public health challenge, which causes antimicrobial resistance and the occurrence of superbugs (Podder et al., 2018; Teng et al., 2020). It not only prolongs treatment but also declines life expectancy due to higher morbidity/mortality risk (Podder et al., 2018; Liu et al., 2020; Estes et al., 2021). These serious public health challenges require the development of new bactericidal methods (Herman and Herman, 2014; Gatadi et al., 2021). With the development of nanotechnology, new antibacterial agents have arisen (Herman and Herman, 2014; Liu et al., 2020). These nanoscale agents may provide more effective and/or more convenient routes (Dizaj et al., 2014; Herman and Herman, 2014). Particularly, nanomaterials based on inorganic metal oxide semiconductors, i.e., CuO, ZnO, MgO, etc., have been considered as alternative antibacterial materials (Zhang et al., 2010; Bafekr and Jalal, 2018; Hong et al., 2018; Chandra et al., 2019; Ogunyemi et al., 2020; Zhang et al., 2010; Baruah et al., 2021; Haider et al., 2021; Han et al., 2021; Qian et al., 2021), whose activities can easily be tuned by altering their morphology and/or component (Prasanna and Vijayaraghavan, 2015).

Generally, reactive oxygen species (ROS) can injure biomolecules by its strong oxidation potential (Sharifi et al., 2012; Prasanna and Vijayaraghavan, 2015; Podder et al., 2018; Yao et al., 2020). The oxidant activity of metal oxide semiconductor nanomaterials usually originates from light-induced oxidative properties to generate ROS, including hydroxyl radicals (·OH), superoxide anions radicals (·O_2_^−^), and singlet oxygen (^1^O_2_) (Podder et al., 2018). The generation of ROS under light exposure comes from the photo-generated electron-hole pairs excited on the appropriate band levels through the absorption of light, which interact with water and then produce ROS (Hirakawa and Nosaka, 2002; Prasanna and Vijayaraghavan, 2015). In addition to photoexcitation, the ROS can also be produced by electrons trapped by the defects at the surface of materials in the absence of light (Prasanna and Vijayaraghavan, 2015; Hao et al., 2017). However, we found that doped agents, which can provide free electrons to induce the production of ROS, also result in the antibacterial activity without external light exposure. MnO_2_ has five different phases (α, β, γ, λ, and δ) and the different properties of each phase make it be extensively studied in the fields of catalysis (Suib, 2008; Truong et al., 2012; Zhou et al., 2017). Moreover, MnO_2_ can effectively enhance the produce of ·OH in the aqueous solution *via* the excitation and formation of electron-hole pairs (Das et al., 2017; Xiao et al., 2018; Chhabra et al., 2019). Particularly, δ-MnO_2_, with a unique layered crystalline structure, has aroused much investigation. By changing the quantity of filling ions between MnO_2_ layers, its doping level can easily be tuned (Golden et al., 1986; Luo et al., 2008; Geng et al., 2016).

We report herein the synthesis of δ-MnO_2_ nanoflowers doped by Mg, Na, and K ions to evaluate the effect of doping on the bacterial viability of *Streptococcus* mutans (S. mutans), a recognized cariogenic bacterium (Song et al., 2020; Afrasiabi et al., 2021). Here, the antibacterial properties of δ-MnO_2_ were probed in the dark (without external illumination). It was observed that all δ-MnO_2_ nanoflowers demonstrated an excellent antibacterial activity without external light exposure. Our data showed that in doped MnO_2_ nanoflowers the free electrons due to doping could induce the production of ROS, resulting in the antibacterial activity. Moreover, δ-MnO_2_ nanoflowers with narrower bandgap displayed a superior antibacterial ability, in which higher doping levels, providing more free electrons to induce the production of ROS, led to better antibacterial properties (following the order: K^+^>Na^+^>Mg^2+^). Furthermore, following the gradual addition of vitamin C, the δ-MnO_2_ could nearly be degraded into Mn ions completely, making this materials potential for *in vivo* applications (Liu et al., 2021).

## Experimental Section

### Materials and Instrumentation

Potassium permanganate (KMnO_4_, >99.5%, Sinopharm), sodium permanganate monohydrate (NaMnO_4_·H_2_O, ≥97%, Sigma-Aldrich), magnesium permanganate hydrate (Mg(MnO_4_)_2_·xH_2_O, Sigma-Aldrich), manganese sulfate monohydrate (MnSO_4_·H_2_O, >99.0%, Sigma-Aldrich), superoxide dismutase (SOD, 15KU, Gunn reagent), vitamin C (VC, 99%, Adamas), and all the chemicals were used without any further purification. De-ionized (DI) water (18.2 MΩ) was used throughout the experiments.

SEM images were obtained by field-emission scanning electron microscopy (FESEM, Hitachi S-4800) which worked at 5 kV. To prepare the samples of SEM, the aqueous suspension including the nanoflowers was dripped on a Si wafer, followed by drying under the air condition. HRTEM images were obtained with a high-resolution transmission electron microscopy (HRTEM, TECHAI G2S-TWIN) operated at 200 kV. To prepare the samples of HRTEM, the alcoholic suspension including the nanoflowers was dripped on a copper grid, followed by drying under the air condition. UV-VIS spectra were obtained from the powder of MnO_2_ with a Shimadzu UV-3600 spectrophotometer. X-ray diffraction (XRD) was characterized by a Rigaku D/Max-2550. X-ray photoelectron spectroscopy (XPS) was carried out using a Thermo Science ESCALAB 250Xi with monochromatic Al Kα (1486.7 eV). The binding energy (BE) was scaled, which regarded the C 1s line at 284.6 eV as the standard for calibration. All data were processed by using the CasaXPS software. The electron spin resonance (ESR) spectra were conducted on a Bruker A300 Electron Paramagnetic Resonance (EPR) Spectrometer.

### Synthesis of MnO_2_ Nanoflowers

MnO_2_ nanoflowers were synthesized through hydrothermal methods as reported previously (Hu et al., 2019; Zhu et al., 2019). For K-doped MnO_2_ nanoflowers, 1.0 g KMnO_4_ and 0.4 g MnSO_4_ were added into the Teflon-lined stainless steel autoclave, together with 30 ml DI water. After stirring for 30 min, the Teflon-lined stainless-steel autoclave was heated and stirred at 140°C for 1 h and then cooled down to room temperature. The obtained MnO_2_ nanoflowers were washed three times with ethanol and three times with DI water by successive cycles of centrifugation and removal of supernatant. Finally, the materials were dried at 60°C for 12 h in a vacuum oven for further use. The Na-doped and Mg-doped samples were prepared with the same procedure, except that the 1.0 g KMnO_4_ was replaced by 1.012 g NaMnO_4_·H_2_O or 0.84 g Mg(MnO_4_)_2_·xH_2_O, respectively.

### Bacteria Culture of *Streptococcus* Mutans

*Streptococcus* mutans (S. mutans) (UA159) were obtained from Shanghai Key Laboratory of Stomatology, Ninth People’s Hospital, Shanghai Jiao Tong University School of Medicine (Shanghai, China). S. mutans was cultured in brain heart infusion broth (BHI broth, Difco laboratories, United States) at 37°C in anaerobic system (N_2_ 80%, H_2_ 10%, CO_2_ 10%). Bacteria were harvested at the exponential growth phase for the use of subsequent experiments.

### Bacterial Viability Test by MTT Assay

The bacterial viability was assessed by 3-[4,5-dimethylthiazol-2-yl]-2,5-diphenyl tetrazolium bromide (MTT) assay. Briefly, S. mutans suspensions at a density of 1 × 10^6^ colony forming units (CFUs)/ml were treated with the different concentrations of Mg-, Na-, and K-doped MnO_2_ nanoflowers (100, 200, 400, and 800 μg/ml) in BHI at 37°C under standard anaerobic conditions (N_2_ 80%, H_2_ 10%, CO_2_ 10%) for 24 h. After 24 h of anaerobic culture, 5 mg/ml MTT solution was added to each well and incubated in dark for 2 h. The supernatant was discarded, and the substrate was reacted in solution by dimethyl sulfoxide (DMSO). The absorbance was tested at 490 nm wavelength using a microplate reader (Bio-Rad, United States). All samples were performed in triplicate. The negative control was the S. mutans group without MnO_2_ nanoflowers treatment. By comparing the OD values (490 nm) of the negative control with that of Mg-, Na-, and K-doped MnO_2_ nanoflowers, the inhibition percentage of bacterial viability was calculated by using the equation: [(OD (negative control)-OD (sample))/OD (negative control)]×100%.

### Biofilm Formation Test by Crystal Violet Assay

Firstly, 1 × 1 cm sterile glass slides were placed in a 24-well plate, and 50 μl ∼10^6^ CFU/ml S. mutans was added to each well. Then, 150 μl suspensions with different concentrations of Mg-, Na-, and K-doped MnO_2_ nanoflowers (100, 200, 400, and 800 μg/ml) were added into the mixture, respectively. In addition, 200 μl ∼10^6^ CFU/ml S. mutans was added to the control wells and anaerobically cultured for 24 h at 37°C. After 24 h of anaerobic culture, the crystal violet was fixed with methanol, stained with 0.1% (w/v) crystal violet, moistened with sterile double steam water, and washed overnight. After drying, the crystal violet was dissolved with 90% ethanol and the absorbance was tested at 550 nm wavelength using a microplate reader (Bio-Rad, United States). All samples were performed in triplicate. The negative control was the biofilm without MnO_2_ nanoflowers. By comparing the OD values (550 nm) of the negative control with that of Mg-, Na-, and K-doped MnO_2_ nanoflowers, the inhibition percentage of biofilm formation was calculated by using the equation: [(OD (negative control)-OD (sample))/OD (growth control)]×100%

### *In vitro* Cytotoxicity Assays

The mouse fibroblast cell line (L929) was obtained from the cell bank of the Chinese Academy of Sciences (Shanghai, China) and cultured in minimum essential medium (Gibco, Life Technologies, Carlsbad, CA) supplemented with 10% fetal bovine serum and 100 U/ml penicillin−streptomycin (Gibco, CA), at 37°C and 5% CO_2_ humidified atmosphere. Cells without any exposure to nanoparticles served as the negative control. Cytotoxicity was assessed by using the MTT assay. Briefly, to evaluate the mitochondrial function and cell viability of L929 cells treated with different concentrations of Mg-, Na-, and K-doped MnO_2_ nanoflowers (100, 200, 400, and 800 μg/ml), cells were seeded at a density of 10^4^ cells/well on 96-well plates and then treated with particles at different concentrations for 24 h. After 24 h treatment, MTT solution (20 μl, 5 mg/ml) (Amersco, Solon, OH, United States) was added into each well and incubated for an additional 4 h in 37°C incubator. Subsequently, 150 μl DMSO was added to dissolve the formazan crystals. The absorbance at 570 and 630 nm was measured by a microplate reader (Multiskan GO, Thermo Scientific, MA, United States).

### ESR Determination

ESR spectroscopy was employed to detect ROS using 5,5-dimethyl-1-pyrroline-N-oxide (DMPO) as a spin trap (Bosnjakovic and Schlick, 2006; Szterk et al., 2011). DMPO traps ·OH to form DMPO−∙OH spin adduct which gives a quadrant signal. It also traps ·O_2_^−^ to form DMPO−·O_2_^−^ spin adduct which also gives a quadrant signal. To suspension of MnO_2_, DMPO was added and the ESR spectra were recorded. All the experiments were performed in dark.

### The Calculation of Atomic Ratios

Take K-doped MnO_2_ nanoflowers as example, the atomic radio between Mn and K was obtained with the quantitative analysis of high-resolution XPS spectra. We regarded I^Mn^ and I^K^ as the Mn and K intensities from one Mn and K atom, respectively. The total intensities of Mn 2p_3/2_ and K 2p_3/2_ can be expressed by following expression (Hu et al., 2019; Zhu et al., 2019):

$${\text{I}}_{\left(\text{TOT}\right)}\left(\text{Mn }2\text{p}\right)=\frac{{\text{I}}^{\text{Mn}}{\text{k}}^{\text{Mn}}}{1-{\text{k}}^{2}}$$ (1)

$${\text{I}}_{\left(\text{TOT}\right)}\left(\text{K }2\text{p}\right)=\frac{{\text{I}}^{\text{K}}{\text{k}}^{\text{K}}}{1-{\text{k}}^{2}}$$ (2)

where the layer-by-layer attenuation factor is given by k = exp (−c/λ sinθ). The c is the depth of atoms, λ is the photoelectron inelastic mean free path, and θ is the takeoff angle relative to the sample surface. Owing to the isotropic property of nanoflowers, k should be integrated to obtain the average. Thus, the layer-by-layer attenuation factor is given by the following expression:

$$\text{k}=\frac{\int_{0}^{\text{π}}\text{exp}\left(-\frac{\text{c}}{\text{λsinθ}}\right)\text{dθ}}{\int_{0}^{\text{π}}\text{dθ}}$$ (3)

The parameter c can be obtained by XRD. The photoelectron inelastic mean free path (λ_Mn_ = 1.73 nm, λ_K_ = 2.25 nm) was calculated by using the National Institute of Standards and Technology (NIST) database.

## Results and Discussion

**FIGURE 1:**
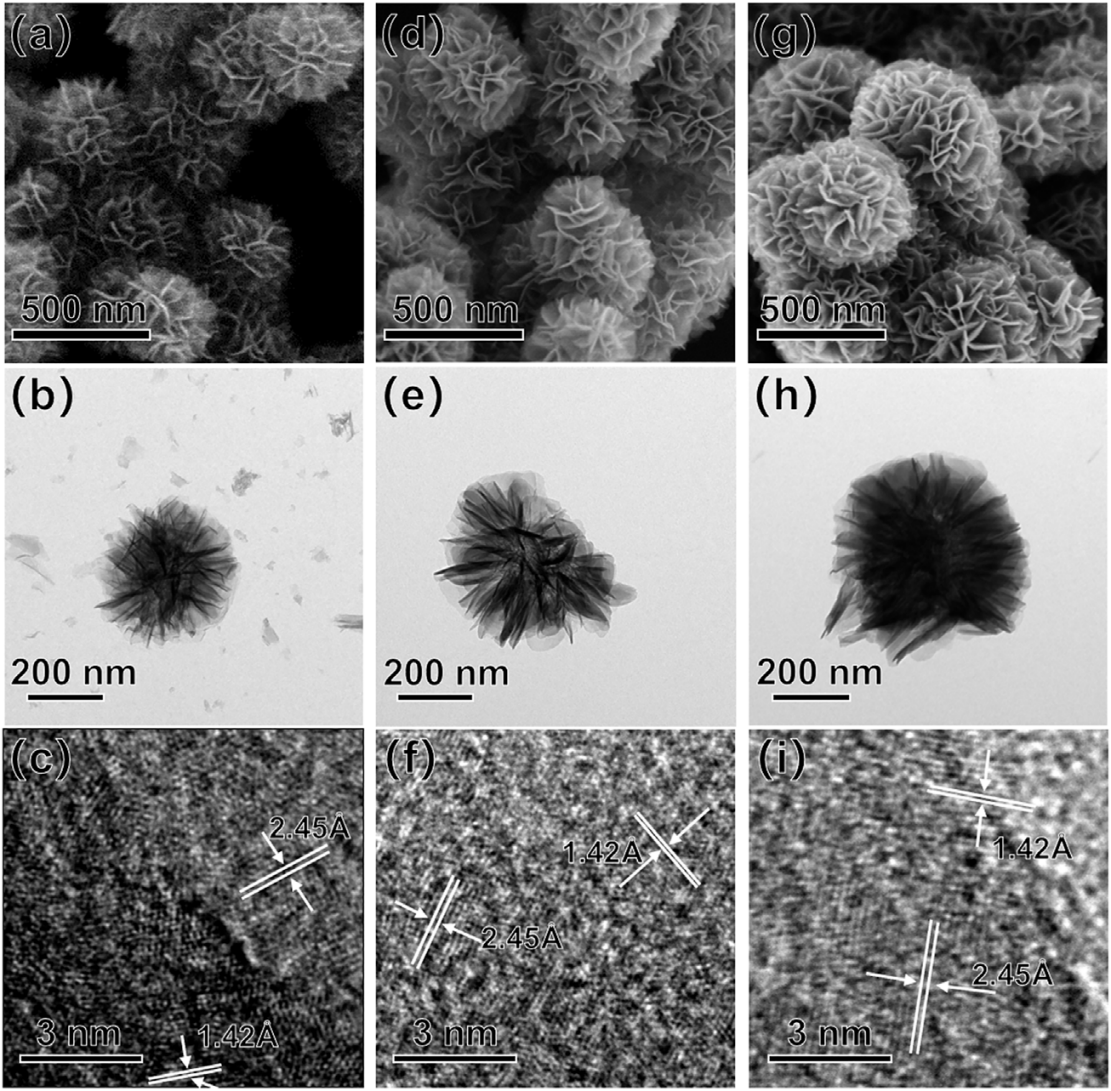
SEM **(A,D,G)**, TEM **(B,E,H)**, and HRTEM **(C,F,I)** images of Mg-, Na-, and K-doped δ-MnO_2_ nanoflowers.

We started by using Mg(MnO_4_)_2_ as precursor to synthesize Mg-doped δ-MnO_2_ materials. Figure 1A shows the SEM image of Mg-doped MnO_2_, which presented a flower-like morphology and the size was ∼380 nm in diameter. Supplementary Figure S1A shows the XRD patterns of Mg-doped δ-MnO_2_ nanoflowers. All the peaks correspond to the crystal planes of δ-MnO_2_ (Hu et al., 2019; Li et al., 2019), and no other crystalline phases were detected. Figures 1B,C present the TEM and HRTEM images of Mg-doped MnO_2_ nanoflowers. The lattice spacings of 1.42 and 2.45 Å coincide with the (110) and (101) interlayer distance in δ-MnO_2_. This is also supported by the SAED patters shown in Supplementary Figure S2A.

The band gap for the Mg-doped MnO_2_ nanoflowers was calculated as 1.13 eV from the UV-VIS spectrum shown in Supplementary Figure S3A. The narrower bandgap observed here relative to the previously reported (Sakai et al., 2005; John et al., 2016) might be attributed to the doping of Mg^2+^ ions between layers of MnO_2_ (Luo et al., 2000; Wang et al., 2017; Hu et al., 2019; Zhu et al., 2019), which will be discussed later.

**FIGURE 2:**
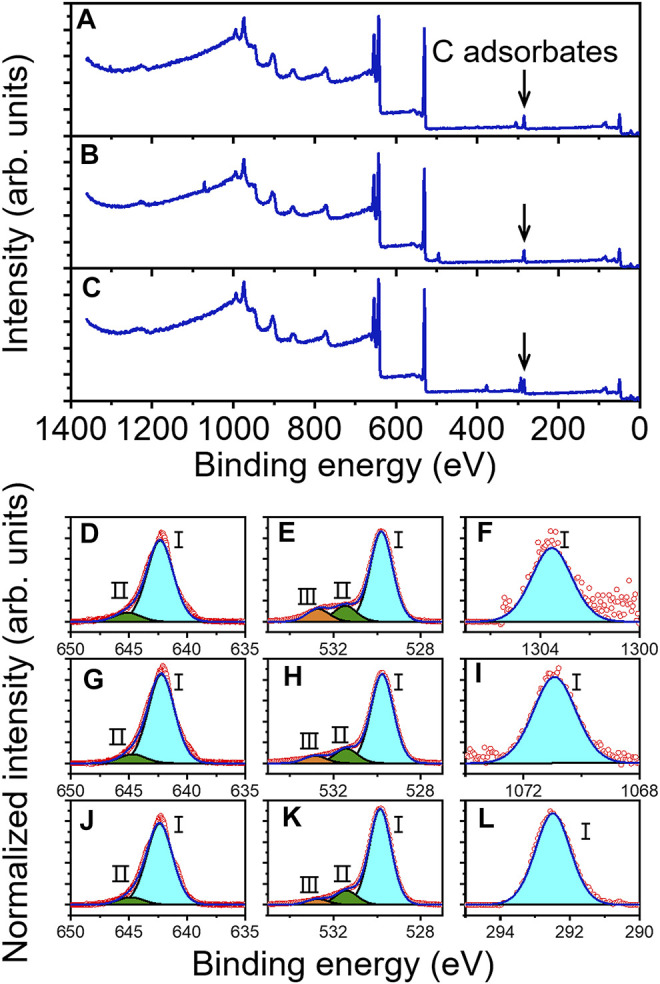
Survey XPS scan of **(A)** Mg-doped, **(B)** Na-doped, and **(C)** K-doped δ-MnO_2_ nanoflowers. Core-level spectra for Mg-doped **(D–F)**, Na-doped **(G–I)**, and K-doped δ-MnO_2_ nanoflowers **(J–L)**: **(D,G,J)** Mn 2p_3/2_, **(E,H,K)** O 1s, **(F)** Mg 1s, **(I)** Na 1s, and **(L)** K 2p_3/2_.

Figure 2A shows the XPS survey spectrum of Mg-doped MnO_2_. It was detected that, with a ∼15 min UV exposure, surface C contamination has a ∼8 times minor intensity relative to that of Mn 2p_3/2_. The binding energy (BE) values of different elements were presented in Supplementary Table S1. The Mn 2p_3/2_ core-level spectrum in Figure 2D presented two components. The main peak labeled I with low BE at 642.3 eV is due to bulk-coordinated Mn, and the other peak with high BE at 645.1 eV (peak II) corresponds to MnO_2_ interacted with absorbed oxygen from air (Selvakumar et al., 2015; Li et al., 2017; Hu et al., 2019; Tang et al., 2020). However, no signal corresponding to reduced Mn^3+^, which is known to occur as a result of the formation of oxygen vacancies, was observed (Selvakumar et al., 2015). Figure 2E shows the O 1s core-level spectrum, which presented three components. The main peak (BE 529.9 eV) labeled I is due to bulk-coordinated oxygen. Peak II (BE 531.5 eV) and peak III (BE 532.9 eV) are attributed to the surface component and the carbonate or hydroxyl groups chemically bound on the surface, respectively (Tang et al., 2020; Arunpandiyan et al., 2021). Figure 2F presents the Mg 1s spectrum which only has one component. The BE of Mg 1s is 1303.5 eV, which is higher than that of Mg_2_Si (∼1302.3 eV) and Mg(OH)_2_ (∼1303 eV) (Esaka et al., 2016; Jiang et al., 2017). The results indicate that Mg^2+^ is chemically bound to MnO_2_ (Nefedov, 1977). In the Mg 1s spectrum only one component was observed, suggesting that all Mg^2+^ ions were filled between the MnO_2_ layers (Hu et al., 2019).

Since the biocompatibility of materials is a prerequisite for their intended use in the human body, *in vitro* cytotoxicity studies of Mg-doped MnO_2_ nanoflowers were carried out prior to antibacterial testing. Specifically, L929 mouse fibroblastic cells were exposed for 24 h to different concentrations (0, 100, 200, 400, and 800 μg/ml) of MnO_2_ nanoflowers and then the cell viability was determined using the MTT assay, a well-established colorimetric method to evaluate the cytotoxicity of biomedical device according to ISO 10993-5:2009. After 24 h of incubation, the cell viability of L929 cells was more than 90% with the concentration of Mg-doped MnO_2_ nanoflowers at 100–400 μg/ml, whereas it was reduced to 66 ± 5.7% when the dose was increased up to 800 μg/ml (Supplementary Figure S4), suggesting that the MnO_2_ nanoflowers exhibited no or low cytotoxicity even at high concentration. When the concentration was higher than 400 μg/ml, an apparent decrease in cell viability was found, indicating high dose of MnO_2_ nanoparticles could induce cell death, which was consistent with our previous studies showing high concentrations of silica nanoparticles could induce cell necrosis in endothelial cells (Liu and Sun, 2010). It might be attributed to the higher cellular uptake of nanoparticles, which could directly damage cell plasma membranes and thus cause cell necrosis (Liu and Sun, 2010).

**FIGURE 3:**
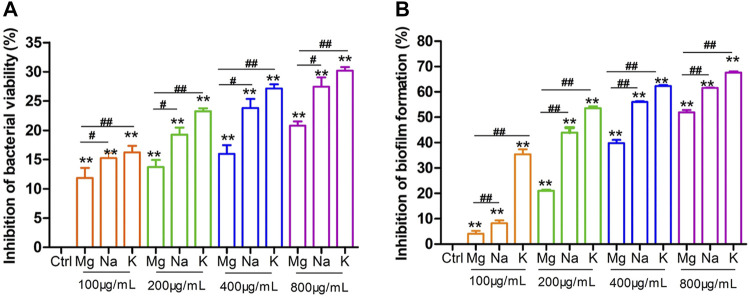
The inhibition effect of Mg-, Na-, and K-doped δ-MnO_2_ nanoflowers on S. mutans bacterial viability and biofilm formation at 24 h. **(A)** Bacterial viability by MTT assay. **(B)** Biofilm formation by crystal violet assay. Bacteria without nanoparticle treatment served as the negative control. Data represents mean ± standard deviation (SD), *n* = 3. **p < 0.01 vs. the negative control group; ##*p* < 0.01 significant difference as compared groups.

Then, the antibacterial activity of Mg-doped MnO_2_ sample was evaluated by detection of the bacterial viability and biofilm formation of S. mutans (Bijle et al., 2020; Daood et al., 2020; Song et al., 2020). Firstly, MTT assay was applied to evaluate the effect of Mg-doped MnO_2_ sample on the bacterial viability of S. mutans. As shown in Figure 3A, Mg-doped MnO_2_ can effectively inhibit bacterial viability. When the Mg-doped MnO_2_ nanoflowers at 100, 200, 400, and 800 μg/ml concentration were added, the percentage of inhibition was 11.86 ± 2.18, 13.73 ± 1.45, 16.0 ± 1.83, and 20.79 ± 0.94%, respectively, reflecting that as the concentration of Mg-doped MnO_2_ nanoflowers increases, the inhibition of bacterial viability shows an upward trend.

Generally, the MTT assay for assessment of antibacterial activity revealed the function of bacterial dehydrogenase system involved in metabolism (He et al., 2015). Furthermore, bacterial biofilm formation is known to increase resistance against the antibiotic and plays a critical role in the pathogenesis of infections (Banerjee et al., 2020). Then, we also examined the effect of Mg-doped MnO_2_ nanoflowers on S. mutans biofilm formation by crystal violet staining (Asgharpour et al., 2019; Chan et al., 2020; Jiang et al., 2020; Song et al., 2020), and the results showed the same trend as the MTT assay (Figure 3B). In the presence of Mg-doped MnO_2_, the inhibition percentage of biofilm formation was 4.1 ± 1.45% at 100 μg/ml, 21.02 ± 0.49% at 200 μg/ml, 39.86 ± 1.59% at 400 μg/ml, and 51.99 ± 1.18% at 800 μg/ml concentration, respectively, showing a dose-dependent enhanced antibacterial activity and reduced biofilm formation against S. mutans induced by Mg-doped MnO_2_ nanoflowers. Interestingly, these antibacterial tests were all performed in the dark.

**FIGURE 4:**
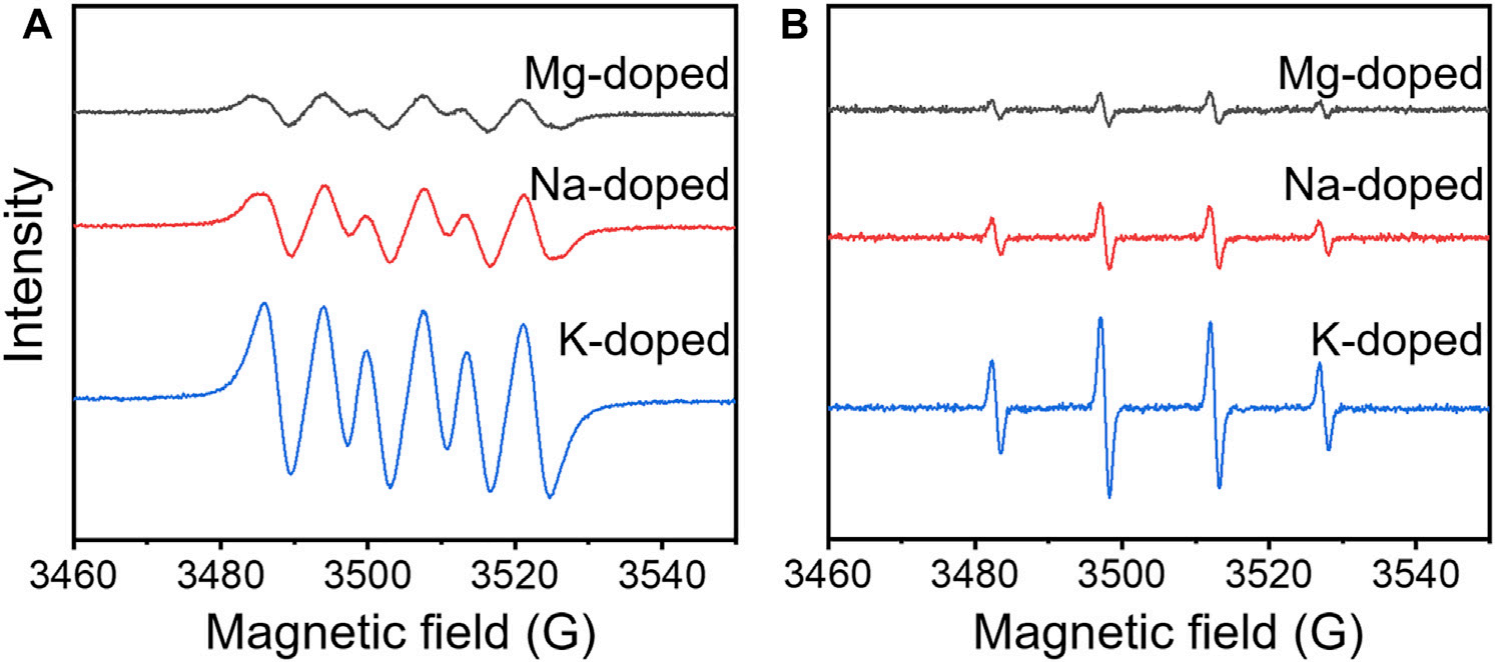
ESR spin trapping spectra of **(A)** DMPO-·O_2_^−^ and **(B)** DMPO-·OH on Mg-, Na-, and K-doped δ-MnO_2_ nanoflowers in the dark.

From the above results, it was intriguing to find that the Mg-doped δ-MnO_2_ nanoflowers exhibited spontaneous antibacterial properties in the dark. Since it is established that ROS is responsible for antibacterial activity in the dark (Prasanna and Vijayaraghavan, 2015), their formation was investigated by electron spin resonance (ESR) using DMPO as a quencher without external illumination. Figures 4A,B show the characteristic DMPO-·O_2_^−^ and DMPO-·OH signals in the case of Mg-doped MnO_2_ nanoflowers at 800 μg/ml concentration (Peng et al., 2021; Zong et al., 2021). The results show that both superoxide radicals (O_2_^−^) and hydroxyl radical ( OH ) were generated under dark conditions.

It has been reported that ZnO and MgO nanoparticles produced ROS in the dark due to the transfer of electrons trapped by the oxygen vacancies at the surface of materials (Prasanna and Vijayaraghavan, 2015; Hao et al., 2017). However, in our case, the presence of oxygen vacancies on the δ-MnO_2_ surface was not detected from the XPS results (Selvakumar et al., 2015). For the Mg 1s core-level spectrum in Figure 2F, it can be observed that Mg is chemically bound to MnO_2_, and thus, the valence electrons of Mg were transferred to MnO_2_, becoming free electrons. It implies that the production of ROS in the dark might be produced by the free electrons in δ-MnO_2_ coming from doping.

In order to further confirm this hypothesis, NaMnO_4_ and KMnO_4_ were employed to synthesize the Na- and K-doped δ-MnO_2_ as similarly described for Mg-doped δ-MnO_2_. In this case, it has been established that these δ-MnO_2_ samples can enable higher doping levels relative to Mg-doped δ-MnO_2_, which can lead to an increase number of free electrons (Hu et al., 2019). Figures 1D,G present their SEM images. These MnO_2_ samples showed similar flower-like morphology as that of Mg-doped ones, and the sizes were ∼460 nm in diameter (Na-doped MnO_2_) and ∼500 nm in diameter (K-doped MnO_2_), respectively. From the XRD patterns in Supplementary Figures S1B,C, it was observed that they all presented very similar crystalline structures to Mg-doped sample. Figures 1E,H present their TEM images. The HRTEM images depicted in Figures 1F,I, as well as SAED patterns in Supplementary Figure S2B,C, display the same lattice spacings (1.42 and 2.45 Å) of MnO_2_ as that of Mg-doped δ-MnO_2_. The bandgaps were calculated from UV-VIS spectra (Supplementary Figures S3B,C, respectively), which were 1.06 (Na-doped MnO_2_) and 0.75 eV (K-doped MnO_2_), respectively.

Figures 2B,C display the XPS survey spectra of Na- and K-doped MnO_2_. In Figure 2I,L, it presented only one component in both Na 1s and K 2p_3/2_ spectra. The BE of Na 1s (1070.9 eV) is higher than that of NaOH (∼1069.6 eV), while the BE of K 2p_3/2_ (292.5 eV) is higher than that of KF (∼292.2 eV) (Oh et al., 2013). Thus, it indicates that Na^+^ or K^+^ ions were also filled between the layers of MnO_2_ (Hu et al., 2019). In Figures 2G,J, Mn 2p_3/2_ core-level spectra presented 2 components, which was consistent with the Mg-doped MnO_2_ sample. The BE of peak II are 644.9 (Na-doped MnO_2_) and 644.7 eV (K-doped MnO_2_), respectively, which are 0.2 and 0.4 eV lower than the corresponding peaks of Mg-doped MnO_2_. Meanwhile, the BE of peak II and III of O 1s peak for Na-doped and K-doped samples also shift to lower BE values relative to those of Mg-doped sample. These variations demonstrate that between MnO_2_ and doped ions, it occurred a charge transfer (Luo et al., 2000; Gupta et al., 2018). The reason can be explained by the different doping levels as follows.

The atomic ratios calculated from the XPS data for Mg/Mn (Mg-doped MnO_2_), Na/Mn (Na-doped MnO_2_), and K/Mn (K-doped MnO_2_) were 1:626, 1:52, and 1:7, respectively. Details on these calculations are described in the experimental section (Hu et al., 2019; Zhu et al., 2019). This difference might be that the bigger size of ions results in larger interaction between ions and MnO_2_ layers, because the ions presented the size of K^+^>Na^+^>Mg^2+^ (Hu et al., 2019). Therefore, the difference in the amounts of doping ions in MnO_2_ results in the variance in the bandgap values, and meanwhile contribute to the lower BE of Mn and O peaks in Na- and K-doped samples relative to those of Mg-doped ones.

Then, MnO_2_ doped by Na^+^ and K^+^ ions were employed to evaluate the effect of free electrons quantity on the bacterial viability and biofilm formation of S. mutans in the dark. *In vitro* cytotoxicity studies were also tested. The experimental procedure was the same as that of Mg-doped MnO_2_ samples, and the results were described in Supplementary Figure S4. Consistent with the cytotoxicity results of Mg-doped MnO_2_, neither Na- nor K-doped MnO_2_ had a significant effect on cell viability at concentration below 400 μg/ml, while a slight reduction in cell viability was observed at 800 μg/ml. Thus, the MTT assay showed no significant cytotoxicity for MnO_2_ against L929 cells when the concentration was no more than 400 μg/ml. It was noted that K-doped MnO_2_ had the least cytotoxicity, while Mg-doped MnO_2_ displayed higher cytotoxicity than Na-doped MnO_2_ sample. According to ISO 10993-5:2009, the biocompatibility of K-doped MnO_2_ was very much within the acceptable limits even at a concentration as high as 800 μg/ml.

Subsequently, we used both the MTT assay and crystal violet staining assay to evaluate the effect of MnO_2_ nanoflowers with different doping on the bacterial viability and biofilm formation of S. mutans. As shown in Figure 3A, the inhibition of S. mutans bacterial viability exposed to Na-doped MnO_2_ nanoflowers at 100, 200, 400, and 800 μg/ml concentration was 15.25 ± 1.11, 19.26 ± 1.39, 23.81 ± 1.83, and 27.46 ± 1.82%, respectively. Meanwhile the inhibition with K-doped MnO_2_ at concentrations of 100, 200, 400, and 800 μg/ml was 16.22 ± 1.38, 23.26 ± 0.65, 27.13 ± 0.98, and 30.20 ± 0.77%, respectively, reflecting that as the concentrations of MnO_2_ nanoflowers increases, the antibacterial activity shows an upward trend.

From Figure 3B, in the presence of Na-doped MnO_2_, the inhibition of biofilm formation was 8.25 ± 1.48% at 100 μg/ml, 44.0 ± 2.40% at 200 μg/ml, 56.0 ± 0.46% at 400 μg/ml, and 61.57 ± 0.24% at 800 μg/ml, respectively. In the presence of K-doped MnO_2_, the inhibition of biofilm formation was 35.44 ± 2.28% at 100 μg/ml, 53.56 ± 1.00% at 200 μg/ml, 62.38 ± 0.46% at 400 μg/ml, and 67.61 ± 0.61% at 800 μg/ml, respectively. Obviously, both MTT assay and crystal violet staining have shown that K-doped MnO_2_ has the superior antibacterial and antibiofilm formation ability, which is better than that of Na-doped sample. Meanwhile, the Mg-doped MnO_2_ had the lowest antibacterial activity. Therefore, these results confirm the hypothesis that higher doping levels could provide more free electrons, which enhanced the antibacterial properties in doped δ-MnO_2_.

Figures 4A,B also show the characteristic DMPO-·O_2_^−^ and DMPO-·OH signals in the case of Na- and K-doped MnO_2_ (Peng et al., 2021; Zong et al., 2021). The results show that both ${\text{O}}_{2}^{-}$ and OH produced in the dark were following the order of K^+^>Na^+^>Mg^2+^, which further confirm that the production of ROS in the dark might be produced by the free electrons in δ-MnO_2_ coming from doping.

**FIGURE 5:**
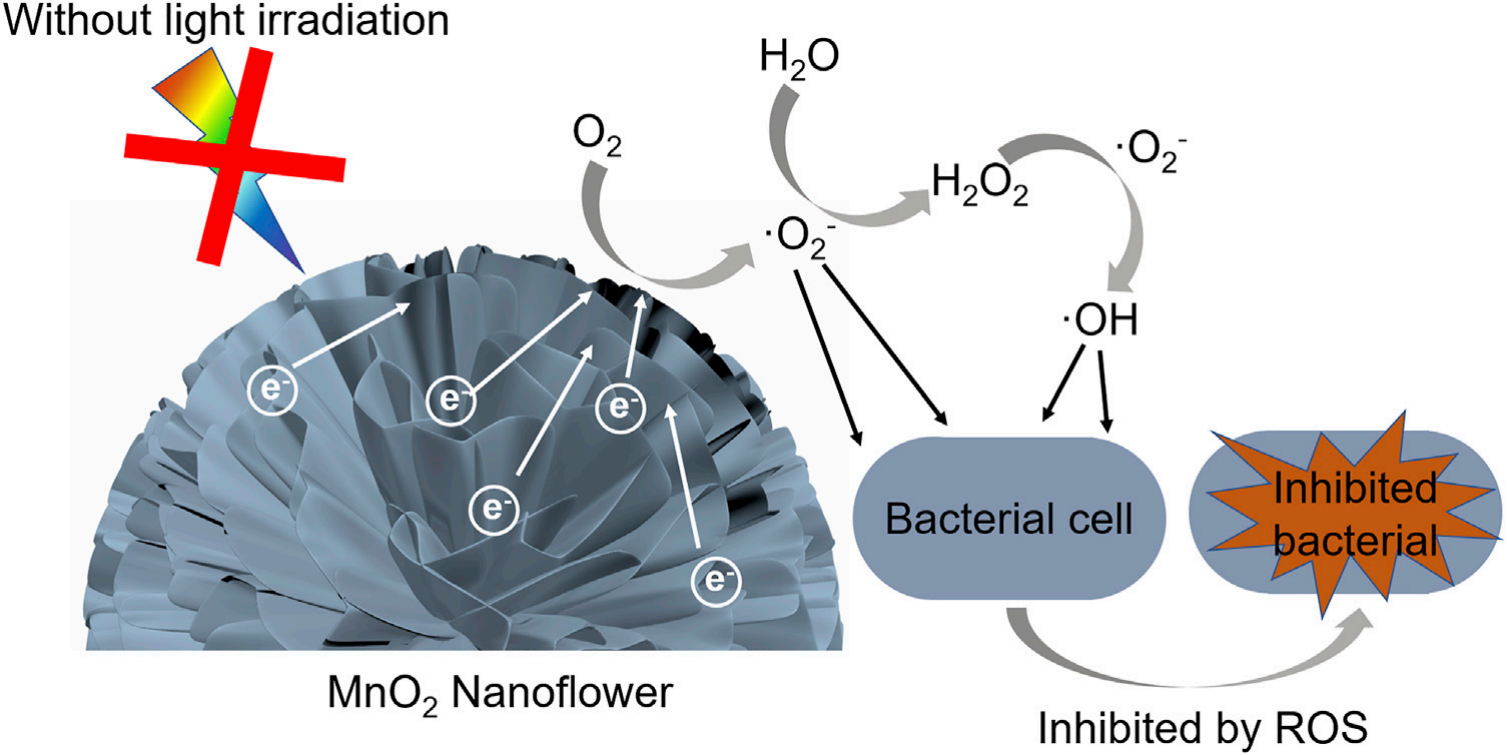
Schematic diagram for the mechanism of antibacterial activity on doped δ-MnO_2_.

In aqueous solutions, the free electrons in δ-MnO_2_ coming from doping can transfer to the water around it and form ·O_2_^−^, while ·OH is known as a derivative of ${\text{O}}_{2}^{-}$ (Morrison et al., 1988; Xu et al., 2020; Yao et al., 2020). To clarify the role of O_2_^−^ in the mechanism of ROS production, ESR was then carried out with the addition of superoxide dismutase (SOD), a well-known superoxide scavenger (Carre et al., 2013; Piccaro et al., 2014; Jiang et al., 2016). The results in Supplementary Figure S5 demonstrate that in the dark both DMPO-·O_2_^−^ and DMPO-·OH signal disappeared in the presence of SOD, revealing that ROS mediated through superoxide plays a major role in antibacterial activity without external illumination (Prasanna and Vijayaraghavan, 2015; Ijaz et al., 2020). Thus, the antibacterial mechanism of doped δ-MnO_2_ could be proposed as in Figure 5. When the alkali metal atoms are chemically bound to MnO_2_ by doping, the valence electrons of alkali metals will transfer to MnO_2_. Then, the doped MnO_2_ can provide free electrons to induce the production of O_2_^−^ in aqueous solutions, which could penetrate into the bacteria cell membrane and then damage cellular components such as DNA and proteins (Xia et al., 2008; Kasemets et al., 2009; Dadi et al., 2019), resulting in the antibacterial activity without external illumination. Furthermore, by comparing the δ-MnO_2_ with different doping level, the ROS generation following the doping levels of K^+^>Na^+^>Mg^2+^ can be observed. With higher doping levels, more free electrons can be transferred to MnO_2_, which induce the production of more ROS, and thus present superior antibacterial activity. Compared to the antibacterial activities of MnO_2_ with other iron oxide nanoparticles such as ZnO, CuO, Fe_3_O_4_, and Al_2_O_3_ (Supplementary Table S2), it was observed that MnO_2_ displayed a superior antibacterial ability.

Supplementary Figure S6 presented the controllable degradation behavior of δ-MnO_2_. Following the gradual addition of vitamin C into the suspension with δ-MnO_2_ samples, it started to fade color, which indicated that MnO_2_ could be degraded in the presence of vitamin C. When the quantity of vitamin C was 14 times over MnO_2_ samples, it observed a nearly complete degradation of MnO_2_ into water soluble Mn ions, which can be rapidly excreted from the body, making this materials potential for *in vivo* applications, presenting not only an outstanding antibacterial efficacy but also an excellent biosafety (Liu et al., 2021).

## Conclusion

δ-MnO_2_ nanoflowers doped by Mg, Na, and K ions were successful synthesized and their bandgap tunable antibacterial properties and controllable degradability mediated by vitamin C were systematically investigated. Interestingly, it was observed that all the samples showed antibacterial activity in the dark, and the antibacterial activity increased with doping levels, which was favored in the K^+^>Na^+^>Mg^2+^ order. Our data suggest that doped MnO_2_ can provide free electrons to induce the production of ROS and result in the antibacterial activity in the dark. Moreover, it is shown that higher doping levels can provide more free electrons, which enhance the antibacterial activity of the δ-MnO_2_ materials. Following the gradual addition of vitamin C, MnO_2_ nanoflowers could nearly be degraded into water soluble Mn ions completely, indicating that these materials also display biosafety. We believe that our results shed light on the design and fabrication of antibacterial nanomaterials with tailored properties.

## Data Availability

The original contributions presented in the study are included in the article/Supplementary Material. Further inquiries can be directed to the corresponding authors.
