# Supplementary material for: Enhanced Spontaneous Antibacterial Activity of δ-MnO2 by Alkali Metals Doping
Source: Front Bioeng Biotechnol. 2022 Jan 4;9:788574. doi: 10.3389/fbioe.2021.788574 (PMC8764136; doi:10.3389/fbioe.2021.788574)
Supplement: Supplementary file 1 [file DataSheet2.pdf]

Table S2 The comparison of antibacterial activities between MnO_2 and other iron oxide (ZnO, CuO, Fe_3O_4, Al_2O_3) nanoparticles.

Nanoparticles	Size (nm)	Morphology	Dosage	Antibacterial Effects	Antibiofilm Effects
**MnO_2**	300~500	Flower-like	100 μg/mL	*S. mutans* (+++)	*S. mutans* (+++)
**ZnO[1]**	50~300	Spherical	100 μg/mL	*S.aureus* (+++)	*S.aureus* (+++)
				P. aeruginosa (+++)	*P. aeruginosa* (+)
				E. coli (++)	*E. coli* (+++)
**CuO[1]**	100~250	Spherical	100 μg/mL	*S.aureus* (++)	*S.aureus* (+)
				P. aeruginosa (+)	*P. aeruginosa* (+)
				E. coli (-)	*E. coli* (+++)
**Al_2O_3[1]**	＜100	Rod-, flake-	100 μg/mL	*S.aureus* (++)	*S.aureus* (-)
				P. aeruginosa (-)	*P. aeruginosa* (-)
				E. coli (-)	*E. coli* (+++)
**Fe_3O_4[1]**	50~150	Cubic shape	100 μg/mL	*S.aureus* (++)	*S.aureus* (+)
				P. aeruginosa (++)	*P. aeruginosa* (-)
				E. coli (++)	*E. coli* (+++)

Note: The antibacterial/antibiofilm effects were defined as follows: high +++, moderate ++, weak +, negative -.

[1] indicates that the data from ref (Sikora et al., 2018).
