# Supplementary material for: Enhanced Spontaneous Antibacterial Activity of δ-MnO2 by Alkali Metals Doping
Source: Front Bioeng Biotechnol. 2022 Jan 4;9:788574. doi: 10.3389/fbioe.2021.788574 (PMC8764136; doi:10.3389/fbioe.2021.788574)
Supplement: Supplementary file 2 [file Image5.pdf]

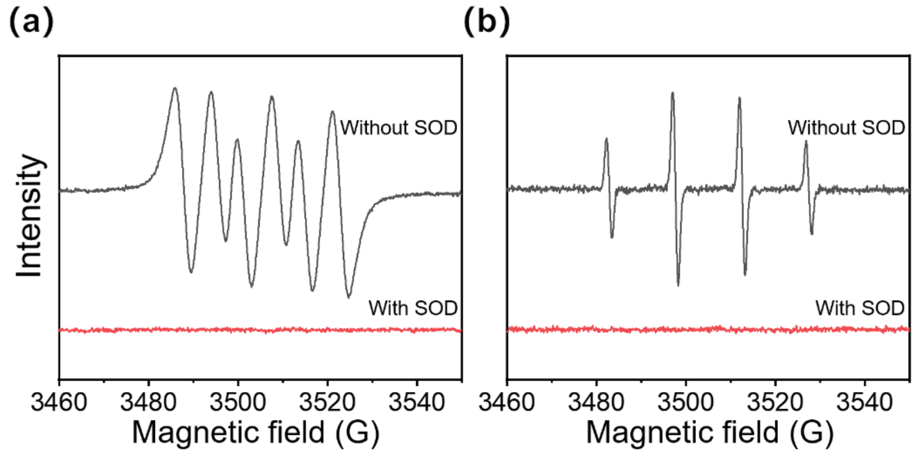

Figure S5. ESR spin trapping spectra of (a) DMPO-$\cdot O_2^-$ and (b) DMPO-$\cdot OH$ on K-doped MnO_2 nanoflowers.
