# Supplementary material for: Enhanced Spontaneous Antibacterial Activity of δ-MnO2 by Alkali Metals Doping
Source: Front Bioeng Biotechnol. 2022 Jan 4;9:788574. doi: 10.3389/fbioe.2021.788574 (PMC8764136; doi:10.3389/fbioe.2021.788574)
Supplement: Supplementary file 3 [file Image6.pdf]

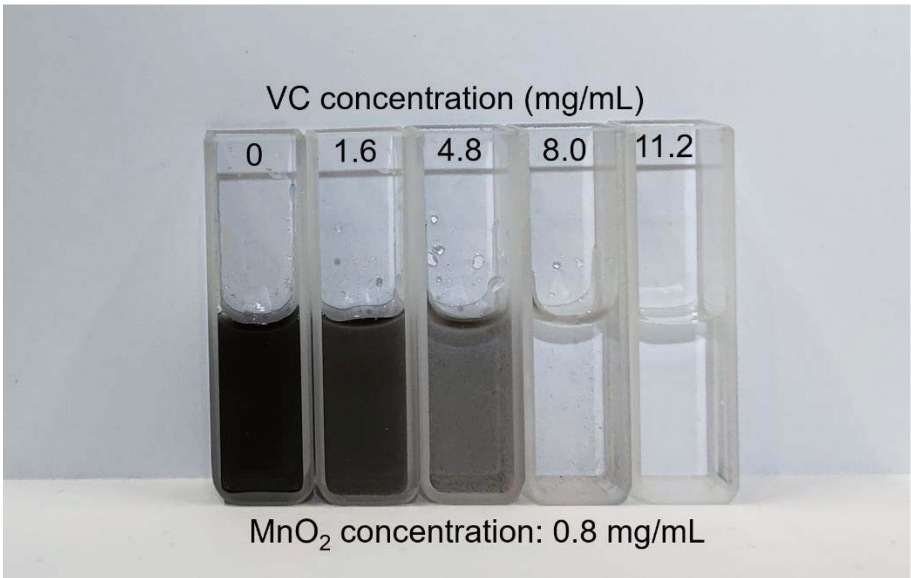

Figure S6. The color change of the solution with 0.8 mg/mL δ-MnO_2 samples following the injection of 0, 1.6, 4.8, 8.0, 11.2 mg/mL Vitamin C, respectively.
