# Supplementary material for: Enhanced Spontaneous Antibacterial Activity of δ-MnO2 by Alkali Metals Doping
Source: Front Bioeng Biotechnol. 2022 Jan 4;9:788574. doi: 10.3389/fbioe.2021.788574 (PMC8764136; doi:10.3389/fbioe.2021.788574)
Supplement: Supplementary file 4 [file Image4.pdf]

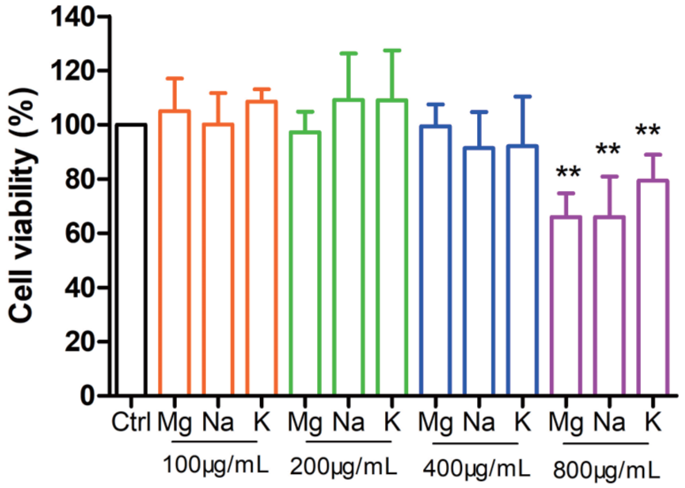

Figure S4. Cytotoxicity measured by the MTT assay for Mg-, Na- and K-doped MnO_2 nanoflowers with different concentration. Normal cells without nanoparticle treatment served as the negative control. Data represents mean ± standard deviation (SD), n= 6. **$p < 0.01$ vs the negative control group.
