# Supplementary figures and images for: Enhanced Spontaneous Antibacterial Activity of δ-MnO2 by Alkali Metals Doping
Source: Front Bioeng Biotechnol. 2022 Jan 4;9:788574. doi: 10.3389/fbioe.2021.788574 (PMC8764136; doi:10.3389/fbioe.2021.788574)

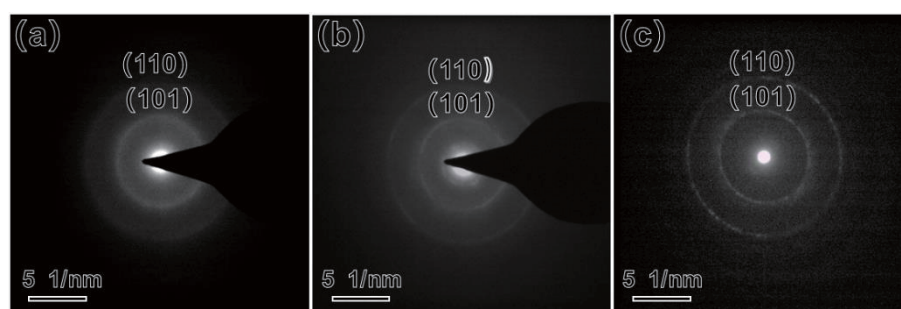

**Figure S2.** SAED patterns for (a) Mg-, (b) Na- and (c) K-doped MnO<sub>2</sub> nanoflowers.

Supplement: Supplementary file 5 [file Image2.pdf]

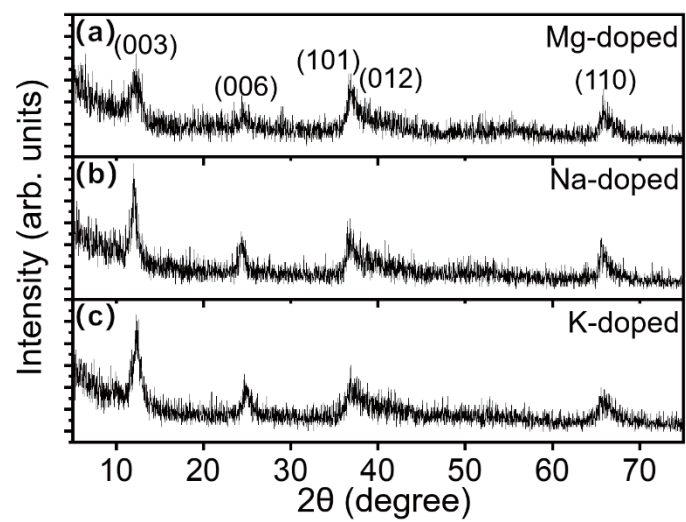

**Figure S1.** XRD patterns of (a) Mg-, (b) Na- and (c) K-doped  $\text{MnO}_2$  nanoflowers.

Supplement: Supplementary file 8 [file Image1.pdf]
