# Supplementary material for: Enhanced Spontaneous Antibacterial Activity of δ-MnO2 by Alkali Metals Doping
Source: Front Bioeng Biotechnol. 2022 Jan 4;9:788574. doi: 10.3389/fbioe.2021.788574 (PMC8764136; doi:10.3389/fbioe.2021.788574)
Supplement: Supplementary file 6 [file Image3.pdf]

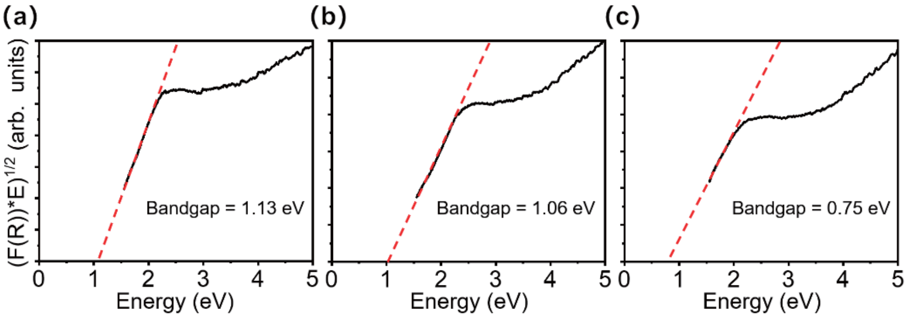

Figure S3. UV-VIS reflection spectra showing the band gaps of (a) Mg-, (b) Na- and (c) K-doped MnO_2 nanoflowers.
