# Supplementary material for: Enhanced Spontaneous Antibacterial Activity of δ-MnO2 by Alkali Metals Doping
Source: Front Bioeng Biotechnol. 2022 Jan 4;9:788574. doi: 10.3389/fbioe.2021.788574 (PMC8764136; doi:10.3389/fbioe.2021.788574)
Supplement: Supplementary file 7 [file DataSheet1.pdf]

Table S1 BE of the Mn $2p_{3/2}$, O 1s, Mg 1s, Na 1s, and K $2p_{3/2}$ core-level components of Mg-, Na-, and K-doped MnO_2 nanoflowers, Respectively.

Component	$Mg-MnO_2$ Peak position (eV)	$Na-MnO_2$ Peak position (eV)	$K-MnO_2$ Peak position (eV)
Mn_{I}	642.3	642.3	642.3
Mn_{II}	645.1	644.9	644.7
O_{I}	529.9	529.9	529.9
O_{II}	531.5	531.4	531.4
O_{III}	532.9	532.8	532.7
Mg	1303.5		
Na		1070.9	
K			292.5
